# Etiology of Out‐of‐Hospital Cardiac Arrest Among Patients Presenting to the Emergency Department of a Tertiary Care Hospital in Bhutan: A Prospective Cohort Study

**DOI:** 10.1155/emmi/8562238

**Published:** 2026-07-22

**Authors:** Ugyen Rinzin, Yeshey Dorjey, Chimi Rinzin, Tsheten Wangchuk

**Affiliations:** ^1^ Department of Emergency Medicine, Eastern Regional Referral Hospital, Mongar, Bhutan, vcu.edu; ^2^ Department of Obstetrics and Gynecology, Eastern Regional Referral Hospital, Mongar, Bhutan; ^3^ Department of Emergency Medicine, Jigme Dorji Wangchuck National Referral Hospital of Bhutan, Thimphu, Bhutan, vcu.edu; ^4^ Department of Surgery, Eastern Regional Referral Hospital, Mongar, Bhutan

**Keywords:** Bhutan, clinical autopsy, etiology, out-of-hospital cardiac arrest

## Abstract

**Background:**

High mortality rates following out‐of‐hospital cardiac arrest (OHCA) make identification of its etiology vital for prevention. In low‐resource settings, limited access to diagnostics and clinical autopsy often prevents definitive conclusions. This study aimed to describe the presumed etiology of OHCA cases presenting to a tertiary emergency department (ED) in Bhutan.

**Methods:**

A prospective cohort study was conducted from August 1, 2023, to July 31, 2024, in the ED of the National Referral Hospital, Bhutan. All patients presenting with OHCA were included and followed until hospital discharge, death, or the end of the study period. Data were extracted from patient records and analyzed using STATA Version 18.

**Results:**

Of the 110 OHCA patients, 59.1% (*n* = 65) were male, and 90.9% (*n* = 100) were adults, with a median age of 53 years (IQR: 32–69). Prehospital emergency medical services (EMS) attended 27.3% (*n* = 30) of cases, and resuscitation was attempted in the ED in 93.6% (*n* = 103). Using the 2024 Utstein OHCA framework, medical causes accounted for 91.8% (*n* = 101) of presumed etiologies and trauma for 6.4% (*n* = 7). Within medical causes, presumed cardiac/unknown was most common (68.3%, *n* = 69), followed by other medical (26.7%, *n* = 27) and respiratory causes (5.0%, *n* = 5). One case each was attributed to drowning/electrocution and asphyxiation (0.9%). Among the 69 presumed cardiac/unknown causes, 94.2% (*n* = 65) had no specific etiology identified after available evaluation, while 5.8% (*n* = 4) had clinical features suggestive of a cardiac etiology. No clinical autopsies were performed. In multivariable logistic regression, increasing age was independently associated with presumed cardiac/unknown with no specific etiology identified after available evaluation (aOR: 1:02; 95% CI: 1.00–1.03; *p* = 0.022), and no other variables were significantly associated with this outcome.

**Conclusion:**

Most OHCA cases were medical in origin, with the majority classified as presumed cardiac/unknown due to the absence of a specific etiology after available evaluation. These findings highlight challenges in etiological classification in resource‐limited settings. Strengthening postresuscitation diagnostic evaluation, standardized post‐ROSC protocols, access to clinical autopsy where feasible, and development of a nationwide OHCA registry may improve etiological classification and guide future preventive strategies.

## 1. Introduction

Out‐of‐hospital cardiac arrest (OHCA) remains a global health priority, with high mortality despite advances in resuscitation science. Survival rates vary significantly by region but remain particularly low in low‐ and middle‐income countries (LMICs) [1, 2]. Over the past decades, efforts have focused on strengthening the “Chain of Survival”—incorporating early recognition, high‐quality cardiopulmonary resuscitation (CPR), timely defibrillation, and structured postcardiac arrest care [3, 4]. While optimizing the resuscitation process is essential, identifying the underlying etiology of OHCA is equally critical for guiding targeted postresuscitation care, informing secondary prevention, and shaping public health interventions. In high‐income countries, acute coronary syndromes (ACS) predominate, though noncardiac causes such as pulmonary embolism, intracranial hemorrhage, and trauma contribute significantly [5]. This etiological spectrum may differ in LMICs due to variations in disease burden, health care access, and diagnostic capacity.

Postresuscitation etiological evaluation among survivors typically includes laboratory testing, electrocardiography, imaging, and a coronary angiogram when indicated. The Utstein reporting template often relies on the classifications of “presumed cardiac” and “obvious non‐cardiac,” which frequently differ from medical or autopsy determinations of etiology [6]. Although autopsy provides definitive etiological data, routine autopsy rates are declining globally and are largely unavailable in many LMIC settings, including Bhutan at the time of the study [7, 8]. Consequently, many OHCA deaths are labeled as presumed cardiac/unknown, hindering the development of targeted preventive strategies.

The 2025 advanced cardiac life support (ACLS) updates emphasize early etiological assessment as a pillar of comprehensive postarrest care, specifically advocating for pan‐computed tomography (CT) to identify reversible causes and resuscitation complications [3]. Despite the global emphasis on improving postcardiac arrest care, the specific etiologies of OHCA in resource‐limited settings remain poorly characterized. This diagnostic gap is exacerbated by limited access to standardized post‐ROSC evaluation bundles. Therefore, the primary objective of this study was to determine the etiological distribution of OHCA, and the secondary objective was to determine the burden of presumed cardiac/unknown etiology with no specific etiology after available evaluation in a setting with a limited post‐ROSC evaluation bundle, in Bhutan.

## 2. Method

### 2.1. Study Design

This was a prospective cohort study conducted over a one year, from August 1, 2023 to July 30, 2024.

### 2.2. Setting and Study Population

The study was conducted in the ED of the National Referral Hospital, a 350‐bed multidisciplinary tertiary care center with 15 clinical departments. The ED provides 24‐hour emergency care to patients of all ages, including medical, trauma, obstetric, and pediatric emergencies, and manages approximately 40,000 visits annually. The study population comprised all patients presenting to the ED with OHCA during the study period. Patients were included irrespective of whether resuscitation was attempted. During the study period, the ED was staffed by 3 emergency specialists, 8 emergency medicine residents, 10 general duty medical officers, 43 nurses, and 6 emergency medical responders. The department operated continuously in three rotating shifts (morning, evening, and night).

### 2.3. Sample Size

Census sampling was utilized for this study; therefore, a formal sample size calculation was not required. Based on retrospective ED triage data, there were 94 documented cases of OHCA in the previous year. To maximize statistical power and eliminate sampling bias, all consecutive OHCA cases presenting during the study period that met the inclusion criteria were included in the final analysis.

### 2.4. Sampling and Recruitment

All consecutive patients presenting to the ED following OHCA during the study period were included. Patients arrived at the ED through three main pathways: (1) transported by family members or bystanders, (2) transported by prehospital emergency medical services (EMS) activated through the national emergency number (112), or (3) referred from lower level hospitals and escorted by healthcare personnel.

Upon arrival, patients were immediately transferred to the resuscitation room and managed according to standard ACLS protocols by the ED code blue team. During resuscitation, arterial blood gas (ABG) analysis was performed when cartridges were available. For patients achieving sustained ROSC, investigations were ordered at the discretion of the treating physician based on clinical judgment and resource availability. Available investigations included electrocardiogram (ECG), renal function and electrolyte testing, complete blood count, liver function test, radiography, blood cultures, bedside ultrasound/point‐of‐care ultrasound (POCUS), echocardiography, CT imaging when clinically indicated, and coronary angiography (CAG) in selected cases. For patients who underwent resuscitation, relevant clinical information was extracted from resuscitation records, clinical notes, and death confirmation documentation after completion of resuscitative efforts. In cases where resuscitation was not initiated or was terminated early, data were obtained from death confirmation records following informed consent obtained through a strict IRB‐approved protocol.

To protect family autonomy during an acute crisis, families were approached for consent only after resuscitation had been completed and the clinical lead had completed bereavement counseling. To avoid perceived coercion, the treating medical staff were excluded from this process; instead, a physician from the Acute Medical Care (AMC) unit with no role in patient care approached the family in a private setting. Because clinical metrics were already captured in routinely documented triage forms, physician notes, and medical records, consent was specifically sought for the secondary research use of these routinely collected data and subsequent follow‐up parameters. Families were briefed on the study’s objectives and were explicitly informed of their right to refuse or withdraw at any point with no impact on patient management.

For patients who achieved return of spontaneous circulation (ROSC) and were admitted to the hospital, in‐hospital outcomes (survival status and documented cause of death) were obtained from admission notes and discharge records. Data collection was performed only after written informed consent had been obtained and resuscitation efforts were completed to ensure that patient care was not interrupted or compromised.

**FIGURE 1:**
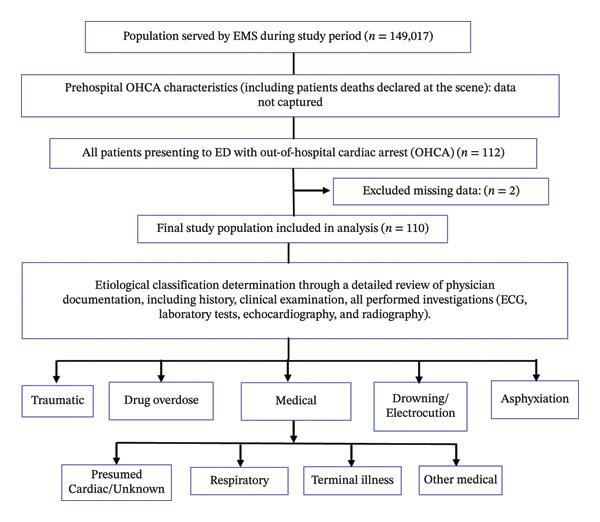
Flow diagram of patient inclusion and presumed OHCA etiology classification according to the 2024 Utstein reporting classifications among patients presenting to the ED of the National Referral Hospital, Bhutan, 2023–2024. (ECG: electrocardiogram; ^∗^Incomplete data missing key clinical data for etiological classification).

Presumed OHCA etiology was classified according to the 2024 Utstein Reporting Recommendations using all available clinical information. Information sources included the prearrest history, circumstances of arrest, physical examination findings, resuscitation records, post‐ROSC laboratory and radiological investigations, and the treating physician’s documented final diagnosis and clinical notes. Patients were followed until discharge or death to capture additional diagnostic information and assign the presumed etiology of OHCA. Cases were classified as medical (presumed cardiac/unknown, respiratory, terminal illness, anaphylaxis, or other medical), trauma, drug overdose, drowning/electrocution, or asphyxia, in accordance with the 2024 Utstein classification. Under the medical category, cases with clinical features suggestive of a cardiac etiology or with no identifiable noncardiac cause after available evaluation were classified as presumed cardiac/unknown. For the secondary analysis, the presumed cardiac/unknown category was further subdivided into cases with clinical features suggestive of a cardiac etiology and those with no specific etiology identified after available evaluation. This exploratory subgroup analysis did not modify the primary Utstein classification. The details are depicted in the flow chart (Figure 1).

### 2.5. Data Collection

Data were collected using a structured paper‐based case record form and extracted from patients’ clinical records. The form was developed in accordance with the study objectives and informed by a review of the relevant literature. Sociodemographic variables included age, sex, occupation, marital status, and mode of transportation to the ED. ED presentation characteristics included mode of presentation (direct arrival or referred from a lower level hospital), resuscitation status in the ED, ED shift of presentation, utilization of prehospital EMS, referral for clinical autopsy, and etiological data classified according to the 2024 Utstein OHCA framework under the subheading Presumed Cause of OHCA.

### 2.6. Data Analysis

Statistical analyses were conducted using STATA Version 18, licensed to the Khesar Gyalpo University of Medical Sciences of Bhutan. Continuous variables were summarized as means or medians, as appropriate, while categorical variables were reported as frequencies and percentages. Univariate logistic regression was performed to examine the association between each independent variable and presumed cardiac/unknown with no specific etiology after available evaluation. Variables with a *p* value < 0.20 in univariable analysis, together with clinically relevant covariates, were entered into the multivariable logistic regression model. The final multivariable logistic regression model demonstrated good calibration (Hosmer–Lemeshow (*χ* ^2^ [8] = 7.07, *p* = 0.529), and no evidence of multicollinearity among the retained predictors (mean variance inflation factor = 1.01), indicating satisfactory model fit and stability. Statistical significance was defined as *p* value < 0.05. The STROBE guidelines were followed in reporting the study findings (Supporting file 1).

## 3. Results

During the study period, a total of 112 patients with OHCA presented to the emergency department (ED). Two patients had incomplete data, with missing key clinical data for etiological classification, and were excluded. Therefore, 110 patients (98.2%) were included in the final analysis, and 103 (93.6%) were resuscitated at the ED.

### 3.1. Sociodemographic Characteristics

A total of 110 OHCA patients were included. The median age was 53 years (IQR: 32–69), and most were adults (90.9%, *n* = 100). Males accounted for 59.1% (*n* = 65). Most patients were married (62.4%, *n* = 63). Over half were unemployed or dependent (52.9%, *n* = 54), followed by private employees (35.3%, *n* = 36). Seasonal distribution was relatively uniform, with summer contributing the highest proportion (31.8%, *n* = 35). More than half of patients arrived by private vehicle (56.1%, *n* = 60), while 30.8% (*n* = 33) used ambulance transport, as shown in Table 1.

**TABLE 1:** Sociodemographic characteristics of OHCA patients presenting to the ED of the National Referral Hospital, Bhutan, 2023‐2024. (*n* = 110).

Characteristics	Number *n* (%)
Age, median (IQR), years	53 (32–69)

*Patient category (n = 110)*	
Pediatric	10 (9.1)
Adults	100 (90.9)

*Sex (n = 110)*	
Male	65 (59.1)
Female	45 (40.9)

*Marital status (n = 101)*	
Married	63 (62.4)
Single	24 (23.7)
Divorced	5 (4.9)
Widowed	9 (8.9)

*Occupation (n = 102)*	
Student	4 (3.9)
Civil servants	4 (3.9)
Private employee	36 (35.3)
Uniformed personnel	1 (0.9)
Monastic body	3 (2.9)
Unemployed or dependent	54 (52.9)

*Season (n = 110)*	
Winter	28 (25.5)
Spring	21 (19.1)
Summer	35 (31.8)
Autumn	26 (23.6)

*Mode of transport (n = 107)*	
Land ambulance	33 (30.8)
Private car	60 (56.1)
Taxi	6 (5.6)
Air ambulance	2 (1.9)
Wheel chair	3 (2.8)
Police vehicle	3 (2.8)

*Note:* IQR: interquartile range; OHCA: out‐of‐hospital cardiac arrest; percentages were based on available case analysis. Missing data: marital status (*n* = 9); occupation (*n* = 8); mode of transport (*n* = 3).

### 3.2. ED Presentation Characteristics

As shown in Table 2, ED arrival times were distributed across shifts: morning 39.1% (*n* = 43), night 33.6% (*n* = 37), and evening 27.3% (*n* = 30). Most patients presented directly to the ED (90.0%, *n* = 99), while 10.0% (*n* = 11) were referred from peripheral hospitals. Prehospital EMS were utilized in 27.3% (*n* = 30) of cases, whereas 72.7% (*n* = 80) had no EMS involvement. The majority of patients (93.6%, *n* = 103) underwent resuscitation in the ED. No postmortem clinical autopsies were performed among the 108 nonsurvivors.

**TABLE 2:** ED presentation characteristics among OHCA patients presenting to the ED of the National Referral Hospital, Bhutan, 2023‐2024. (*n* = 110).

ED characteristics	Number *n* (%)
*Shift duty*	
Morning	43 (39.1)
Evening	30 (27.3)
Night	37 (33.6)

*Mode of presentation*	
Direct presentation	99 (90.0)
Referred in from peripheral hospitals	11 (10.0)

Availed prehospital EMS*^∗^*	
Yes	30 (27.3)
No	80 (72.7)

*Resuscitation attempted at ED*	
Yes	103 (93.6)
No	7 (6.4)

*Referred for autopsy (n = 108* ^∗∗^)	
No	108 (100.0)

Abbreviations: ED = emergency department, EMS = emergency medical service.

^∗^Patients who arrived via ambulance without prehospital EMS personnel (*n* = 3) were categorized as not having received prehospital EMS.

^∗∗^Two survivors were excluded.

### 3.3. Presumed Etiology of OHCA

According to the 2024 Utstein classification framework for presumed OHCA etiology, medical causes were the predominant category, accounting for 91.8% of cases (*n* = 101; 95% CI: 85.2–95.6). Within the medical category, presumed cardiac/unknown etiology was the most common subgroup, representing 68.3% of medical cases (*n* = 69; 95% CI: 58.7–76.6). This was primarily driven by cases in which no specific etiology was identified after available evaluation (*n* = 65, 94.2%; 95% CI: 86.0–97.7), whereas only four patients (5.8%; 95% CI: 2.3–14.0) had clinical features suggestive of a cardiac etiology.

Among the remaining medical causes, respiratory causes accounted for 5.0% of medical cases (*n* = 5, 95% CI: 2.1–11.1). Within the other medical causes subgroup, the most frequent etiologies were septic shock (25.9%, n = 7; 95% CI: 13.2–44.2) and hyperkalemia (25.9%, n = 7; 95% CI: 13.2–44.2), followed by upper gastrointestinal bleeding (18.5% n = 5). Less common causes included subarachnoid hemorrhage (7.4%, n = 2), high‐risk pulmonary embolism (7.4%, n = 2), and spontaneous intracerebral hemorrhage, diabetic ketoacidosis, aspiration pneumonia, acute gastroenteritis, and ruptured ectopic pregnancy, each accounting for 3.7% (n = 1).

Trauma accounted for 6.4% of OHCA cases (n = 7; 95% CI: 3.1–12.6). Among trauma cases, blunt trauma, predominantly resulting in traumatic brain injury, was the most common mechanism accounting for 71.4% (n = 5; 95% CI: 35.9–91.8), while penetrating trauma accounted for the remaining 28.6% (n = 2; 95% CI: 8.2–64.1). The remaining nonmedical causes comprised one case each of drowning/electrocution and asphyxiation due to hanging (0.9% of all OHCA cases), as shown in Table 3.

**TABLE 3:** Presumed etiology of OHCA according to the 2024 Utstein classification among patients presenting to the ED of the National Referral Hospital, Bhutan, 2023–2024 (*n* = 110).

Utstein category of presumed etiology	Number *n* (%)	95% CI
Medical	101 (91.8)	85.2–95.6
Presumed cardiac/unknown	69 (68.3)	58.7–76.6
Respiratory	5 (5.0)	2.1–11.1
Anaphylaxis	0 (0.0)	0.0–3.6
Terminal illness	0 (0.0)	0.0–3.6
Other medical	27 (26.7)	19.0–36.2
Trauma	7 (6.4)	3.1–12.6
Blunt	5 (71.4)	35.9–91.8
Penetrating	2 (28.6)	8.2–64.1
Burn injury	0 (0.0)	0.0–35.4
Drug overdose	0 (0.0)	0.0–3.3
Drowning/electrocution	1 (0.9)	0.2–4.9
Asphyxia	1 (0.9)	0.2–4.9
Hanging	1 (100.0)	20.7–100.0
Medical subgroup: presumed cardiac/unknown (*n* = 69)		
Clinical features suggestive of a cardiac etiology	4 (5.8)	2.3–14.0
No specific etiology after available evaluation	65 (94.2)	86.0–97.7
Medical subgroup: respiratory (*n* = 5)		
Hypoxia	4 (80.0)	37.6–96.4
Aspiration pneumonia	1 (20.0)	3.6–62.4
Medical subgroup: other medical (*n* = 27)		
Septic shock	7 (25.9)	13.2–44.2
Hyperkalemia	7 (25.9)	13.2–44.2
Upper gastrointestinal bleeding	5 (18.5)	8.2–36.7
Subarachnoid hemorrhage	2 (7.4)	2.1–23.4
High‐risk pulmonary embolism	2 (7.4)	2.1–23.4
Spontaneous intracerebral hemorrhage	1 (3.7)	0.7–18.3
Diabetic ketoacidosis	1 (3.7)	0.7–18.3
Acute gastroenteritis	1 (3.7)	0.7–18.3
Ruptured ectopic pregnancy	1 (3.7)	0.7–18.3

*Note:* Presumed cardiac etiology includes cases with clinical features suggestive of cardiac causes as well as cases in which no alternative noncardiac cause was identified after available evaluation, in accordance with the 2024 Utstein OHCA reporting framework.

### 3.4. Factors Associated With Presumed Cardiac/Unknown With No Specific Etiology After Available Evaluation

Under the 2024 Utstein reporting framework, OHCA cases with clinical features suggestive of a cardiac cause, as well as cases in which no specific noncardiac etiology can be identified after available evaluation, are classified as presumed cardiac/unknown. In our cohort, presumed cardiac/unknown comprised 68.3% (*n* = 69) of medical etiologies. To better characterize this heterogeneous group, we performed an exploratory subgroup analysis by subdividing the presumed cardiac/unknown category into two mutually exclusive subgroups: (1) cases with clinical features suggestive of a cardiac etiology and (2) cases with no specific etiology identified after the available evaluation. This subgroup analysis was performed solely for analytical purposes and does not represent a modification of the Utstein reporting classification.

Among the 69 patients classified as presumed cardiac/unknown, 94.2% (*n* = 65) had no identifiable etiology after the available evaluation. Whereas 5.8% (*n* = 4) had clinical features suggestive of a cardiac cause. Logistic regression analysis was subsequently performed to identify factors associated with presumed cardiac/unknown with no identifiable etiology after the available evaluation. After adjustment, increasing age remained independently associated with presumed cardiac/unknown with no identifiable etiology after the available evaluation (aOR: 1.02; 95% CI: 1.00–1.03; *p* = 0.022), corresponding to a 2% increase in the odds for each 1‐year increase in age. Although patients referred from peripheral hospitals were less likely to have no identifiable etiology after the available evaluation in the univariable analysis (cOR: 0.22; 95% CI: 0.06–0.89; *p* = 0.035), this association was attenuated, after adjustment it did not reach statistical significance (aOR: 0.25; 95% CI: 0.06–1.04; *p* = 0.058) as shown in Table 4.

**TABLE 4:** Factors associated with presumed cardiac/unknown with no identifiable etiology after the available evaluation among OHCA patients presenting to the ED of the National Referral Hospital, Bhutan, 20232–024 (*n* = 110).

Factors	cOR (95% CI)	*p* value	aOR (95% CI)	*p* value
Age (per year increase)^∗^	1.01 (1.00–1.03)	0.013	1.02 (1.00–1.03)	0.022

*Sex*				
Male	1.00 [Ref]	—	—	—
Female	1.46 (0.67–3.19)	0.343	—	—

*Mode of presentation*				
Direct presentation	1.00 [Ref]	—	1.00 [Ref]	—
Referrals from peripheral hospitals	0.22 (0.06–0.89)	0.035	0.25 (0.06–1.04)	0.058

*Availed prehospital EMS*				
No	1.00 [Ref]	—	—	—
Yes	0.60 (0.26–1.39)	0.237	—	—

*Note:* IQR: interquartile range; Ref: references.

Abbreviations: aOR = adjusted odds ratio, cOR = crude odds ratio, EMS = emergency medical service.

^∗^Age presented as median (IQR).

## 4. Discussion

Our study highlights a substantial diagnostic gap in determining the etiology of OHCA in Bhutan’s tertiary care ED, as 94.2%(*n* = 65) of patients classified as presumed cardiac/unknown had no specific etiology identified after available evaluation. This proportion is markedly higher than that reported internationally, where only 11%–16% of OHCA cases remain unexplained [9, 10]. In high‐resource settings, standardized postresuscitation protocols and routine clinical autopsies enable near‐complete etiological classification [11, 12]. Additionally, alternative approaches such as verbal autopsy, commonly used in LMICs, were not implemented in our setting. Collectively, these gaps may contribute to the high proportion of presumed cardiac/unknown without a specific etiology after available evaluation. This highlights the potential value of structured post‐ROSC evaluation protocols and routine clinical autopsy services to better inform future prevention strategies.

An etiology was identified in 40.9% of cases classified as trauma, drowning/electrocution, asphyxia, and other medical causes, suggesting that potentially reversible causes may be underrecognized. Presumed cardiac causes accounted for 62.7% (*n* = 69) of all OHCA cases, comparable to international reports ranging from 36% to 87.9% [11, 13–15]. However, the 2024 Utstein framework classifies cases with no specific etiology identified after available evaluation as presumed cardiac etiology by default [16]. To better characterize this category, we further subdivided presumed cardiac/unknown cases into those with clinical features suggestive of cardiac etiology and those with no specific etiology identified after available evaluation. We found that 94.3% (*n* = 65) of presumed cardiac cases belonged to the latter group. This may suggest that many OHCA cases are classified as presumed cardiac by default rather than because of evidence supporting a cardiac cause, potentially obscuring the burden of other etiologies and limiting opportunities for targeted prevention. This challenge is compounded by the absence of clinical autopsies, as previous reviews have demonstrated inaccuracies in etiological classification using the Utstein framework when compared with clinical autopsy findings [6]. Strengthening etiological evaluation is therefore essential to improve classification accuracy and inform prevention strategies.

In our study, only one patient underwent CAG following ROSC, consistent with current guidelines advocating a selective approach [17]. However, outcomes after OHCA are known to be better in CAG‐capable centers, highlighting the importance of improving access to advanced cardiac diagnostic services as part of comprehensive postresuscitation care [18].

Among identified medical causes, other medical etiologies predominated, including hyperkalemia (25.9%, *n* = 7), septic shock (25.9%, *n* = 7), and upper gastrointestinal bleeding (18.5%, *n* = 5). These likely represent only a fraction of the true disease burden because etiological classification relied on available clinical documentation and investigations rather than standardized diagnostic protocols. Limited use of post‐ROSC pan‐CT may also have contributed to lower identification of noncardiac causes such as pulmonary embolism, aortic dissection, and occult hemorrhage, although this study did not directly evaluate the incremental diagnostic yield of imaging modalities. Previous studies have demonstrated that early imaging can assist in identifying such conditions [11], and the 2025 American Heart Association (AHA) guidelines support consideration of pan‐CT in post‐ROSC care [19].

In our setting, etiological determination largely relied on prearrest history, circumstances of arrest, physical examination findings, resuscitation records, post‐ROSC laboratory and radiological investigations, and the treating physician’s documented final diagnosis and clinical notes. Consequently, diagnostic consistency may have varied depending on the individual’s judgment and experience. In the absence of routine autopsy and pan‐CT, the distinction between noncardiac medical etiologies from presumed cardiac cases with no specific etiology identified after available evaluation remains subjective. Standardized post‐ROSC protocols incorporating investigations such as ABG, POCUS, and pan‐CT may improve etiological identification and should be explored in future system‐level initiatives. For example, the observed prevalence of hyperkalemia (*n* = 7) is likely underestimated, due to inconsistent access to ABG testing, often limited by cartridge shortages. Consequently, fatal arrhythmias secondary to renal failure may remain unrecognized, particularly given prior local data demonstrating high OHCA incidence and mortality among patients with end‐stage renal disease [20].

The high proportion of presumed cardiac cases with no specific etiology identified after available evaluation poses a major barrier to effective public health prioritization. Implementing an evidence‐based postresuscitation diagnostic framework, integrating advanced imaging, standardized laboratory evaluation, and clinical autopsy for nonsurvivors, is, therefore, a public health necessity in Bhutan. Although establishing clinical autopsy services requires long‐term investment in training and infrastructure, accurate etiological data are essential to guide prevention and strengthen resuscitation systems. In resource‐limited settings, OHCA may be underrecognized as a public health priority, and many patients are transported to hospitals by private means rather than formal EMS. In our study, a large proportion (56.1%, *n* = 60) of OHCA cases were brought to the hospital by private vehicles, suggesting that many community OHCA events may never reach healthcare facilities and that the true burden is likely underrepresented. Nevertheless, each OHCA case represents an important opportunity to identify potentially modifiable risk factors.

A standardized verbal autopsy may serve as a pragmatic interim strategy to reduce the proportion of presumed cardiac/unknown cases with no specific etiology identified after available evaluation and bridge the existing data gap in Bhutan [21, 22]. In current practice, information gathering in the ED is brief and targeted, similar to other emergency presentations. This may have contributed to a higher proportion of presumed cardiac/unknown cases with no specific etiology, particularly among patients who were not resuscitated or did not achieve sustained ROSC, where further investigations were limited. Adopting a standardized approach, such as the World Health Organization (WHO) verbal autopsy framework [23] for all OHCA presentations, may reduce the proportion of presumed cardiac cases with no specific etiologies after available evaluation and better inform preventive public health measures.

Additionally, increasing age was independently associated with presumed cardiac disease, with no specific etiology identified after available evaluation. However, given the modest effect size, small sample size of (*n* = 110) and limited number of patients with identified etiology (*n* = 45), this finding has limited clinical relevance and should be interpreted cautiously. No other variables were significantly associated with this outcome. These findings highlight the need for a nationwide OHCA registry to enable improved etiological classification and support evidence‐based prevention strategies.

### 4.1. Limitations

This study has several limitations. First, this was a single‐center study conducted in a resource‐limited setting; therefore, the findings may not be generalizable to other healthcare systems or populations. In addition, no formal a priori sample size calculation was performed. Instead, all consecutive eligible patients presenting during the study period were included, which may have limited the statistical power of subgroup and multivariable analyses. Second, etiological classification was based on physician documentation, available clinical information, and investigations, without routine clinical autopsy or standardized advanced diagnostic evaluation. Consequently, misclassification and interobserver variability cannot be excluded. The high proportion of presumed cardiac cases with no specific etiologies (94.2%) likely reflects these diagnostic constraints.

Although the 2024 Utstein framework classifies cases without an identified nonexternal cause as presumed cardiac/unknown, we further subdivided this category into cases with clinical features suggestive of cardiac etiology and those with no specific etiology identified after available evaluation. This was done based on a comprehensive review while remaining consistent with the Utstein framework, although it may reduce direct comparability with studies strictly adhering to standard Utstein reporting. Third, our study included only patients who reached the ED, excluding those who died at the scene or during transport, introducing potential selection bias. Important prehospital variables, including initial rhythm, bystander CPR, and EMS response times, were unavailable because of the developing prehospital EMS system and inconsistent prehospital documentation. This limited adjustment for important confounders may reduce the internal validity of the multivariable analysis. Therefore, residual confounding cannot be entirely ruled out, and our findings should be interpreted as strong clinical associations rather than direct causal relationships. Furthermore, patients achieving sustained ROSC underwent more extensive diagnostic evaluation than those who died early or did not undergo resuscitation. This may introduce potential survival and classification bias, and these factors may have contributed to the high proportion of cases presumed cardiac with no specific etiology identified after available evaluation.

## 5. Conclusion

The high proportion of OHCAs with presumed cardiac with no specific etiologies after available evaluation (94.2%) in this single‐center study may reflect limitations in the completeness of diagnostic evaluation following OHCA in this resource‐limited setting. Given the limited availability of confirmatory investigations and standardized postresuscitation assessment, the reported etiological patterns should be interpreted with caution. Nevertheless, these findings identify potential areas for system improvement, including development of a nationwide OHCA registry, standardized post‐ROSC evaluation protocols, improved access to diagnostic imaging, and consideration of routine clinical autopsy services where feasible. Such measures may support improved etiological classification and better understanding of OHCA patterns in low‐resource settings.

## Author Contributions

Ugyen Rinzin, Yeshey Dorjey, Chimi Rinzin, and Tsheten Wangchuk: conceptualization, data collection, methodology, writing–original draft, and writing–review and editing.

## Funding

The authors have nothing to report.

## Ethics Statement

The research was conducted following approval from the Institutional Review Board of the Khesar Gyalpo University of Medical Sciences of Bhutan, IRB/Approval/PN/2022/034/564. Additionally, the investigation was carried out in adherence to the ethical standards outlined in the 1964 Declaration of Helsinki and its subsequent amendments or comparable ethical standards. Written informed consent was obtained from a guardian or family. They expressed their willingness to permit the utilization of clinical data and the disclosure of this information for academic purposes. This consent specifies that all information capable of identifying or correlating to the individual will be anonymized to ensure their confidentiality.

## Consent

Please see the Ethics Statement.

## Conflicts of Interest

The authors declare no conflicts of interest.

## Supporting Information

Additional supporting information can be found online in the Supporting Information section.

## Supporting information


**Supporting Information** The STROBE guidelines were followed in reporting the study findings (Supporting file 1).

## Data Availability

The data examined in the present study can be obtained from the corresponding author upon reasonable request.

## References

[1] Berdowski J. , Berg R. A. , Tijssen J. G. P. , and Koster R. W. , Global Incidences of Out-of-Hospital Cardiac Arrest and Survival Rates: Systematic Review of 67 Prospective Studies, Resuscitation. (November 2010) 81, no. 11, 1479–1487, 10.1016/j.resuscitation.2010.08.006. 20828914

[2] Kiguchi T. , Okubo M. , Nishiyama C. et al., Out-Of-Hospital Cardiac Arrest Across the World: First Report from the International Liaison Committee on Resuscitation (ILCOR), Resuscitation. (July 2020) 152, 39–49, 10.1016/j.resuscitation.2020.02.044. 32272235

[3] Executive Summary: 2025 International Liaison Committee on Resuscitation Consensus on Science with Treatment Recommendations, Circulation. https://www.ahajournals.org/doi/10.1161/CIR.0000000000001361. 10.1161/CIR.0000000000001361 41122844

[4] Del R. M. , Bartos J. A. , Panchal A. R. et al., Part 1: Executive Summary: 2025 American Heart Association Guidelines for Cardiopulmonary Resuscitation and Emergency Cardiovascular Care, Circulation. (2025) 152, no. 2, S284–S312, 10.1161/CIR.0000000000001372. 41122893

[5] Myat A. , Song K. J. , and Rea T. , Out-of-Hospital Cardiac Arrest: Current Concepts, The Lancet. (March 2018) 391, no. 10124, 970–979, 10.1016/S0140-6736(18)30472-0. 29536861

[6] Shaeri S. , Considine J. , Dainty K. N. , Olasveengen T. M. , and Morrison L. J. , Accuracy of Etiological Classification of Out-of-Hospital Cardiac Arrest: A Scoping Review, Resuscitation. (May 2024) 198, 10.1016/j.resuscitation.2024.110199. 38582438

[7] Khan M. A. and Verma M. , Benefits and Future of Clinical Autopsy: A Literature Review, Cureus. (2025) 17, no. 9, 10.7759/cureus.93412. PMC12560806 41164059

[8] Tseng Z. H. , Olgin J. E. , Vittinghoff E. et al., Prospective Countywide Surveillance and Autopsy Characterization of Sudden Cardiac Death, Circulation. (June 2018) 137, no. 25, 2689–2700, 10.1161/CIRCULATIONAHA.117.033427. 29915095 PMC6013842

[9] Tabi M. , Perel N. , Taha L. et al., Out of Hospital Cardiac Arrest-New Insights and a Call for a Worldwide Registry and Guidelines, BMC Emergency Medicine. (August 2024) 24, no. 1, 10.1186/s12873-024-01060-4. PMC11297571 39095722

[10] Wittwer M. R. , Zeitz C. , Beltrame J. F. , and Arstall M. A. , Aetiology of Resuscitated Out-of-Hospital Cardiac Arrest Treated at Hospital, Resuscitation. (January 2022) 170, 178–183, 10.1016/j.resuscitation.2021.11.035. 34871757

[11] Branch K. R. H. , Gatewood M. O. , Kudenchuk P. J. et al., Diagnostic Yield, Safety, and Outcomes of Head-to-Pelvis Sudden Death CT Imaging in Post Arrest Care: The CT FIRST Cohort Study, Resuscitation. (July 2023) 188, 10.1016/j.resuscitation.2023.109785. 37019352

[12] Yoshimura S. , Tseng Z. H. , Yamada T. et al., Underlying Cause of Out‐of‐Hospital Cardiac Arrests in Japan in Survivors Versus Nonsurvivors, Journal of the American Heart Association. (May 2025) 14, no. 9, 10.1161/JAHA.124.036968. PMC12184230 40240947

[13] Ramaka S. , Nazir N. T. , Murthy V. S. et al., Epidemiology of Out-of-Hospital Cardiac Arrests, Knowledge of Cardiovascular Disease and Risk Factors in a Regional Setting in India: The Warangal Area Out-of-Hospital Cardiac Arrest Registry (WACAR), Indian Heart Journal. (November 2020) 72, no. 6, 517–523, 10.1016/j.ihj.2020.10.002. 33357639 PMC7772591

[14] Claesson A. , Djarv T. , Nordberg P. et al., Medical Versus Non Medical Etiology in Out-of-Hospital Cardiac Arrest-Changes in Outcome in Relation to the Revised Utstein Template, Resuscitation. (January 2017) 110, 48–55, 10.1016/j.resuscitation.2016.10.019. 27826118

[15] Bardai A. , Berdowski J. , van der Werf C. et al., Incidence, Causes, and Outcomes of Out-of-Hospital Cardiac Arrest in Children: A Comprehensive, Prospective, Population-Based Study in the Netherlands, Journal of the American College of Cardiology. (May 2011) 57, no. 18, 1822–1828, 10.1016/j.jacc.2010.11.054. 21527156

[16] Cardiac Arrest and Cardiopulmonary Resuscitation Outcome Reports: 2024 Update of the Utstein Out-of-Hospital Cardiac Arrest Registry Template, 10.1161/CIR.0000000000001243. 39045606

[17] Wańha W. , Kołodziejczak M. , Kowalewski M. et al., Out-of-Hospital Cardiac Arrest: Do We Have to Perform Coronary Angiography?, Cardiology Journal. (December 2023) 30, no. 6, 1026–1037, 10.5603/CJ.a2023.0032. 37183538 PMC10713207

[18] Ebrahimian S. , Coaston T. , Vadlakonda A. et al., Outcomes of Out‐Of‐Hospital Cardiac Arrest at Centers With and Without On‐Site Coronary Angiography: A Nationwide Analysis, Journal of the American Heart Association. (July 2025) 14, no. 13, 10.1161/JAHA.125.042369. PMC12449992 40530473

[19] Hirsch K. G. , Amorim E. , Coppler P. J. et al., Part 11: Post–Cardiac Arrest Care: 2025 American Heart Association Guidelines for Cardiopulmonary Resuscitation and Emergency Cardiovascular Care, Circulation. (October 2025) 152, no. 2, S673–S718, 10.1161/CIR.0000000000001375. 41122894

[20] Rinzin U. , LeVine S. , Watts M. , Tshering U. , and Tshering K. , Frequency of ED Visits and Mortality Among the Adults With ESRD on Twice-Weekly Maintenance Hemodialysis at Tertiary Care Hospital in Bhutan, International Journal of Emergency Medicine. (August 2025) 18, no. 1, 10.1186/s12245-025-00894-4. PMC12392477 40877755

[21] Thomas L. M. , D’Ambruoso L. , and Balabanova D. , Verbal Autopsy in Health Policy and Systems: A Literature Review, BMJ Global Health. (May 2018) 3, no. 2, 10.1136/bmjgh-2017-000639. PMC5935163 29736271

[22] Bailo P. , Gibelli F. , Ricci G. , and Sirignano A. , Verbal Autopsy as a Tool for Defining Causes of Death in Specific Healthcare Contexts: Study of Applicability Through a Traditional Literature Review, International Journal of Environmental Research and Public Health. (September 2022) 19, no. 18, 10.3390/ijerph191811749. PMC9517079 36142022

[23] World Health Organization , Verbal Autopsy Standards: 2022 WHO Verbal Autopsy Instrument, 2025, World Health Organization, Geneva, https://www.who.int/publications/m/item/training-curriculum-for-the-training-of-verbal-autopsy-master-trainers-and-supervisors.

